# Resistant Starch Consumption Effects on Glycemic Control and Glycemic Variability in Patients with Type 2 Diabetes: A Randomized Crossover Study

**DOI:** 10.3390/nu13114052

**Published:** 2021-11-12

**Authors:** Yolanda Arias-Córdova, Jorge Luis Ble-Castillo, Carlos García-Vázquez, Viridiana Olvera-Hernández, Meztli Ramos-García, Adrián Navarrete-Cortes, Guadalupe Jiménez-Domínguez, Isela Esther Juárez-Rojop, Carlos Alfonso Tovilla-Zárate, Mirian Carolina Martínez-López, José D. Méndez

**Affiliations:** 1 Centro de Investigación, División Académica de Ciencias de la Salud (DACS), Universidad Juárez Autónoma de Tabasco (UJAT), Villahermosa 86150, Mexico; yolandaariasc@gmail.com (Y.A.-C.); gbasecs@hotmail.com (C.G.-V.); viryolvera11@gmail.com (V.O.-H.); meztli.garcia@hotmail.com (M.R.-G.); iselajuarezrojop@hotmail.com (I.E.J.-R.); martinezlopez@hotmail.com (M.C.M.-L.); 2 Departamento de Medicina Interna, Hospital General de Zona No. 46, Instituto Mexicano del Seguro Social (IMSS), Villahermosa 86060, Mexico; adrianavarrete@msn.com (A.N.-C.); jimenezg03@hotmail.com (G.J.-D.); 3 División Académica Multidisciplinaria de Comalcalco (DAMC), Universidad Juárez Autónoma de Tabasco (UJAT), Comalcalco 86650, Mexico; alfonso_tovillaz@yahoo.com.mx; 4 Hospital de Cardiología, Centro Médico Nacional Siglo XXI, Instituto Mexicano del Seguro Social (IMSS), Ciudad de México 06703, Mexico; mendezf@unam.mx

**Keywords:** resistant starch, banana, glycemic variability, glycemic control, type 2 diabetes, continuous glucose monitoring

## Abstract

We previously observed beneficial effects of native banana starch (NBS) with a high resistant starch (RS) content on glycemic response in lean and obese participants. Here, we aimed to determine the effects of NBS and high-amylose maize starch (HMS) on glycemic control (GC) and glycemic variability (GV) in patients with type 2 diabetes (T2D) when treatments were matched for digestible starch content. In a randomized, crossover study, continuous glucose monitoring (CGM) was performed in 17 participants (aged 28–65 years, BMI ≥ 25 kg/m^2^, both genders) consuming HMS, NBS, or digestible maize starch (DMS) for 4 days. HMS and NBS induced an increase in 24 h mean blood glucose during days 2 to 4 (*p* < 0.05). CONGA, GRADE, and J-index values were higher in HMS compared with DMS only at day 4 (*p* < 0.05). Yet, NBS intake provoked a reduction in fasting glycemia changes from baseline compared with DMS (*p* = 0.0074). In conclusion, under the experimental conditions, RS from two sources did not improve GC or GV. Future longer studies are needed to determine whether these findings were affected by a different baseline microbiota or other environmental factors.

## 1. Introduction

The prevalence of type 2 diabetes (T2D) is rising worldwide, and Mexico is classified among the top 10 countries for number of adults (20–79 years) with 15.2% prevalence and 38.6% undiagnosed cases [1]. Uncontrolled T2D is associated with long-term microvascular complications, such as nephropathy, neuropathy, and retinopathy, as well as cardiovascular disease [2]. Particularly, glycemic variability (GV), understood as upward and downward acute glycemia fluctuations within a determined range, is associated with oxidative stress damage to cells [3]. Thus, strict glucose management is crucial in order to prevent microvascular complications in these patients. Among the strategies to achieve this goal is the adoption of healthy behaviors such as dietary changes and regular physical activity.

In this line, the quality of carbohydrates in the diet has been considered an important factor for modulating the rate of glucose absorption and modifying the postprandial glycemic response [4,5]. Available carbohydrates are those that are digested and absorbed and are considered glucogenic in humans. These substances are defined as the sum of free sugars (glucose, fructose, sucrose, maltose, lactose, and oligosaccharides) and complex carbohydrates (dextrins, starch, and glycogen). In most plant-based foods, the major contributor to available carbohydrates is digestible starch (DS). This has been subcategorized as rapidly digested starch (RDS) and slowly digested starch (SDS) according to the incubation time for hydrolysis by α-amylase and amyloglucosidase [6]. Resistant starch (RS) is considered a fermentable fiber that can resist digestion in the stomach and small intestine and reaches the large intestine mainly undigested, and is fermented by the intestinal microbiota [7]. Among the different types of RS, RS type 2 (RS2) refers to starch molecules with type B or C polymorph that can come from different sources like uncooked potato, green banana, and high-amylose corn. Some of the proposed mechanisms of these substances for reducing glycemic response are the capacity to form viscous solutions, delay gastric emptying, and/or inhibiting lipase activity [8]. Most studies discussing the benefits of RS2 have been performed using a commercial ingredient high-amylose maize starch (Hi-Maize^®^), which is isolated from a special hybrid of corn that is naturally high in amylose content [9]. Few studies have investigated the effects of other RS2 obtained from other sources such as barley, brown rice, beans, or green bananas.

Native banana starch (NBS) obtained from unripe ‘Dwarf Cavendish’ bananas (*Musa acuminata*, AAA Group) is rich in RS2 and has been studied by our group. For instance, long-term NBS supplementation has been proven to have beneficial effects in participants with obesity [10,11]. Moreover, a single acute administration induced a reduction in glycemic and insulin response in healthy participants [12]. In another study, the administration of 40 g of RS2, distributed into two beverages/day for four days to lean and obese participants, reduced the 48 h glycemia AUC, but there were no modifications in GV indexes [13]. However, we attributed these findings to the lack of altered GV among the studied group. Although RS2 has exhibited potential beneficial effects in healthy persons or patients with obesity, nowadays, their effects on GV in T2D subjects have been less investigated.

Recently, the usage of systems for continuous glucose monitoring (CGM) is increasing rapidly around the world, particularly in patients with T2D. This tool provides much more accurate information including magnitude, duration, and frequency of glycemia fluctuations. It can be used over several days in patients in a real-world setting, offering more advantages than the simple fasting glycemia or the oral glucose tolerance test.

Therefore, the aim of the present study was to compare the effects of RS from two sources on glycemic control (GC) and GV assessed by CGM in patients with T2D when all treatments were matched by digestible starch content. We hypothesized that RS supplementation would improve GC and reduce GV indexes.

## 2. Materials and Methods

### 2.1. Participants

Seventeen participants were recruited among patients attending regular medical consultations at the General Hospital of the Instituto Mexicano del Seguro Social (IMSS) from February 2016 to April 2017. Participants of both genders were included if they had a previous diagnosis of T2D from a health care provider of the IMSS, had glycosylated hemoglobin (HbA1c) values >6.5%, were younger than 65 years old, were under medical treatment with diet and exercise, or used metformin and/or glibenclamide for glycemic control (without dose modification during the experimental period). Study participation did not limit the medical management of patients supplied by health care providers. Participants not included in this study were those who had complications such as cardiovascular, renal, hepatic, or gastrointestinal diseases (Crohn’s disease, colitis, gastroenteritis, celiac disease, short bowel syndrome); those who were under treatment with insulin, glitazones, DPP4 inhibitors, GLP-1 analogues, or other drugs that alter the absorption of monosaccharides such as acarbose; those who were under some form of psychiatric treatment; those who were pregnant; those who had habits of alcoholism or smoking; those who practiced physical exercise for more than 4 h a week; and those who had non-permeable veins.

The experimental protocol was approved by the Ethical Committee of the IMSS under registration number 2015-2701-17 in compliance with the ethical principles and guidelines of the Declaration of Helsinki for the protection of human subjects under research. This trial was registered at www.anzctr.org.au (ACTRN12621001382864). Written informed consent was obtained from all patients.

### 2.2. Starch Sources

Bananas (*Musa acuminata*, AAA Group, ‘Dwarf Cavendish’) were purchased from a packing plant in Villahermosa in the State of Tabasco, Mexico. The bananas were of a first to second degree of ripeness. The isolation procedure for banana starch was performed according to Waliszewski et al. with slight modifications. Bananas were washed and rinsed with a 0.5% citric acid solution to reduce their oxidation. The peel was removed, sliced 3–5 cm thick, and dipped immediately into the same solution. Fruit pieces were macerated at a low speed in an industrial blender (10 kg fruit/10 L of solution) for 4 min. The homogenate was washed three times through filtration (40-, 60-, and 100-mesh screen) with a 0.3% citric acid solution. The filtrate was sedimented for 16 h at 4 °C and the supernatant was separated by decantation. The sediment was dried at 50 °C in a convection oven (Model SEM-2, Mapisa Internacional Polinox, Ciudad de México, Mexico) for 16 h, ground in a mill (Model 80393, Hamilton Beach^®^, Inc., Glen Allen, VA, USA), sieved (100 mesh), and stored at 4 °C in a sealed plastic container [14]. The RS content of NBS was 70.5% according to Megazyme commercial kit K-DSTRS 11/2019 (Megazyme Ltd. Co, Wicklow, Ireland). Hi-Maize^®^ 260 (high amylose maize starch) containing 60% RS and 40% digestible starch (DS) and Amioca^®^ containing 0% RS and 100% DS were purchased from Ingredion Mexico, S.A. de C.V (Guadalajara, Mexico).

### 2.3. Study Design and Protocol

**Figure 1:**
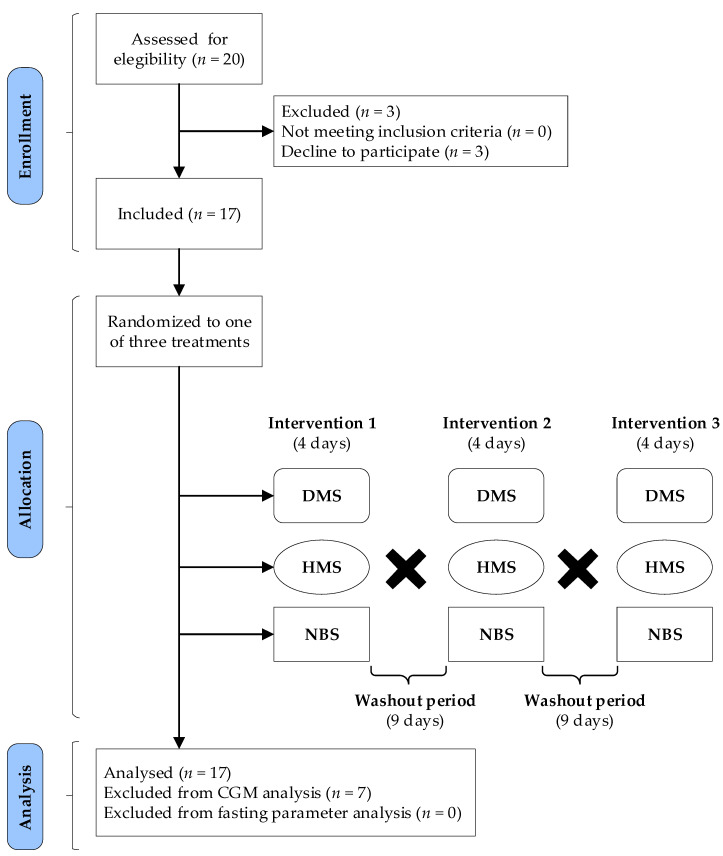
Flow diagram of participants in enrollment, allocation, analysis, and experimental design. A total of 17 volunteers were randomly allocated to receive digestible maize starch (DMS), high-amylose maize starch (HMS), or native banana starch (NBS) during 4 days’ intervention. The black crosses represent the crossover design in which the participants crossover from one treatment to another after 9-day washout periods.

The study was a randomized, crossover, single-blind controlled design (Figure 1). One week before the experimental period, a nutritionist provided detailed dietary advice for establishing the standard size of food portions so that participants could provide a daily food intake record during the intervention days. A daily food record format was given to the participants on the first day of each treatment phase to provide the type and amount of consumed food during days 1 to 4. Moreover, ingestion of medications was also recorded, specifying the exact time they were administered. In the first treatment phase, participants were asked to maintain their habitual dietary intake, but with a restriction in dietary fiber consumption (under 15 g/day, without considering the treatments fiber content). During the second and third treatment phases, patients maintained the same food intake pattern to achieve a similar caloric intake in all treatment phases. The daily energy intake, macronutrients, and dietary fiber were calculated according to the Mexican System of Food Equivalents (Sistema Mexicano de Alimentos Equivalentes) [15].

The experimental period comprised three treatment phases, each with a duration of 4 days, and 9-day washout periods between treatments (Figure 1). On day 1, at 07:00, fasting samples were taken and the Enlite^®^ sensor (Medtronic, Inc., Northridge, CA, USA) was subcutaneously inserted for registering the CGM. Then, the beverages containing the different treatments began to be consumed. For calibrations, participants completed four blood glucose meter readings per day using the FreeStyle system from Abbott Laboratories. From days 1 to 4, each participant consumed two daily beverages containing one of three different supplements for the randomly assigned treatment. A computer-based online random number generator was used to assign participants to the respective treatments. On day 5 of each experimental period, blood samples were obtained after 10 h fasting state and, at 15:00, the sensor was removed.

### 2.4. Treatments

The DMS group received 26.6 g/day of Amioca^®^ (100% DS). The HMS group received 66.6 g/day of Hi-Maize^®^ 260 (60% RS and 40% DS) to provide 26.6 g of DS and 40 g of RS. The NBS group received a combination of 57.2 g/day of NBS and 20.92 g/day of Amioca^®^ to provide 26.6 g of DS and 40 g of RS, based on the content of 70.5% RS and 10% DS. Thus, all supplements were matched in terms of digestible starch content [16] and the RS contents were similar in both the HMS and NBS groups.

The total daily dose of each treatment was divided into two portions provided to the participants in individual sachets. Participants were instructed to mix the test starches with a drink and ingest them twice a day, one at breakfast (07:00–09:00) and another at lunch (13:00–15:00) during the first 4 days of each treatment phase. In all cases, adherence to treatment was monitored by personal phone calls and requiring the participants to return treatment containers on day 5 of each treatment phase. The symptoms of intolerance to supplements were also estimated.

### 2.5. Continuous Glucose Monitoring

GV and GC were determined using the MiniMed iPro^®^ 2 CGM system (Medtronic MiniMed, Northridge, CA, USA), which consists of the Enlite^®^ sensor from Medtronic, which was subcutaneously inserted into the periumbilical area, recording an interstitial glucose reading every 5 min (for a total of 288 reads over a 24 h period per day) during the treatment phase. After using the sensor, the participants returned the recorder to upload the data to a web-based software, which provided a summary of the glucose response. To calculate the CGM measures for the purposes of this study, we considered all the calibrated glucose profiles recorded in the patients’ database over all the experimental periods.

To estimate GC, we used the 24 h mean blood glucose (24 h MBG), which is an indicator of mean daily glycemia, as well as the high blood glycemic index (HBGI) and low blood glycemic index (LBGI), which are typically employed to visualize CGM traces in risk space. Large amplitude of glycemic excursions (LAGE) was calculated as the difference between the maximum and minimum glucose levels. TAR was defined as the percent time with glucose levels above 180 mg/dL; TIR was defined as the percent time with glucose levels between 70 mg/dL and 180 mg/dL; and TBR was defined as the percent time with glucose levels less than 70 mg/dL [17]. All these parameters account for the frequency and amplitude of hyperglycemic or hypoglycemic events.

In this study, as indexes of GV, we considered the coefficient of variation (CV), which refers to the ratio of SD to MBG; continuous overlapping net glycemic action (CONGA), which represents the SD of the glycemic changes recorded between a specific point on the CGM profile and a point 𝑛 hours earlier; glycemic risk assessment in diabetes equation (GRADE), which summarizes the degree of risk associated with a glucose profile; mean absolute glucose (MAG), which is the summed differences between sequential glucose profiles per 24 h divided by the time in hours between the first and last glucose measurement; J-Index, defined as the square of the mean plus SD of glucose measurements, excluding severe and persistent hypoglycemia; mean amplitude of glucose excursion (MAGE), calculated as the arithmetical mean of the differences between consecutive glycemic peaks and nadirs, reflecting major glucose fluctuations of more than 1 SD in the glycemic values; and mean of daily differences (MODD) measures inter-day GV, which reflects the stability of day-to-day blood glucose patterns [18,19].

### 2.6. Biochemical Determinations

Blood samples were centrifuged, and the serum was separated for the determination of glucose, cholesterol, triglycerides, GLP-1, and insulin. Samples that were not immediately analyzed were stored at −70 °C until further analysis. Glucose, cholesterol, and triglycerides determinations were performed using Abbott’s Architect c8000 Clinical Chemistry Autoanalyzer. GLP-1 was determined by an enzymatic-linked immunosorbent assay (Millipore Corporation Pharmaceuticals, St. Charles, MO, USA). Insulin was measured by immunoassay of chemiluminescent microparticles (CMIA) using an INMULITE 1000 System (Siemens Medical Solutions Diagnostics, Los Angeles, CA, USA). HbA1c was determined using the D-10 Hemoglobin Testing System from Bio-Rad. Insulin resistance (IR) at fasting was estimated according to the homeostatic model assessment (HOMA-IR), calculated as the product of fasting glucose (mg/dL) and insulin (μU/mL) divided by 405 [20].

### 2.7. Statistical Analysis

In order to detect a difference of 10% in the primary outcome variable of 24 h MBG with a power >0.8 and a type I error of 5% in a crossover design, a group of 10 participants was used. Data from the CGM were revised in the CareLink™ iPro software (Medtronic Inc., Northridge, CA, USA) and four capillary glucose calibrations per day were integrated before being downloaded and exported to Excel. To determine the different GV indexes, EasyGV 6.0 software was used. Time-course data were analyzed by two-way repeated measures ANOVA to assess the effects of treatment, time, and the interaction of treatment and time. One-way repeated measures ANOVA or Student’s *t*-tests were used to compare the effects of treatments on fasting parameters or GV indexes. Incremental glucose values during CGM were calculated by subtracting concentrations at time = 0 from those at the following time points. Data were expressed as mean ± standard deviation (SD), unless otherwise specified. The D’Agostino–Pearson normality test was performed to assess whether the data were consistent with a Gaussian distribution. Differences were considered statistically significant at *p* < 0.05. Data were processed and analyzed using the GraphPad Prism Software version 7.0 (San Diego, CA, USA).

## 3. Results

### 3.1. Participant Characteristics

The baseline characteristics of the patients are shown in Table 1. Twenty participants were recruited; however, three of them withdrew from the study because of a lack of adherence to treatments. Seventeen patients (9 men and 8 women) completed the study for the fasting parameters’ evaluation; however, only 10 of them exhibited complete CGM data along the study. All patients exhibited uncontrolled glycemia with HbA1c levels over 6.5%. Most of the patients (70.0%) were overweight or obese according to World Health Organization (WHO) criteria (BMI values ≥ 25). Sixty percent of participants were under metformin and glibenclamide treatment, and 40% were under metformin. In general, all starch supplements were well tolerated. However, after ingesting NBS, one patient (1/10) reported moderate abdominal discomfort with flatulence and abdominal bloating. According to the crossover design used, each participant received all treatments (DMS, HMS, and NBS); the order of treatments was randomly assigned.

### 3.2. Dietary Intake

Table 2 shows the average daily dietary intake in patients during the three treatment phases of the experimental period. There were not significant differences in daily caloric intake, macronutrients, and fiber among treatments, time, or in the interaction between treatment and time.

### 3.3. Effects of Treatments on Glycemic Control

The CGM analysis was performed on data from 10 patients who had all their glycemia values registered through days 2 to 4. Data from the first day were excluded owing to missing values and unstable measurements. During days 2 to 4, HMS and NBS exhibited higher 24 h mean blood glucose (24 h MBG) values in comparison with DMS (*p* = 0.0001). On day 2, no differences were observed between HMS and NBS; however, on days 3 and 4, HMS had higher values than NBS (*p* = 0.0001) (Table 3). Considering that DMS had lower baseline glycemia values than HMS and NBS, we decided to analyze incremental 24 h MBG values profile: on day 2, HMS had higher values with respect to DMS (*p* = 0.0001) and NBS (*p* = 0.0001); on day 3, HMS and NBS had higher values than DMS (*p* = 0.0001). No differences were observed between HMS and NBS on days 2 and 3. On day 4, NBS showed higher values than DMS and HMS (*p* = 0.005, *p* = 0.0016, respectively). A graphical presentation of the glycemic profiles during CGM is shown in Supplementary Material, Figures S1−S3. The LBGI values were not different between groups during all 3 days of CGM. However, on day 4, the HBGI in the HMS group exhibited higher values compared with DMS (5.37 (2.20, 24.16) vs. 3.17 (0.49, 9.79), *p* = 0.002) (Table 3). There were no significant differences in maximum and minimum glucose, LAGE, TAR, TBR, and TIR values between the three groups during the 3 days. Nonetheless, the NBS treatment displayed a trend of provoking higher TAR values (48.09%) with respect to DMS or HMS (7.98% and 25.52%, respectively).

### 3.4. Effects of Treatments on Glycemic Variability

In relation to GV, no differences between groups were observed during days 2 and 3; however, on day 4, higher GV indexes were observed in the HMS group compared with DMS: CONGA (7.44 (6.47, 13.58) vs. 6.65 (5.88, 9.98), *p* = 0.04); J-Index (35.76 (24.36, 82.68) vs. 27.90 (16.53, 45.60), *p* = 0.011); and GRADE (10.80 ± 8.85 vs. 6.65 ± 7.08, *p* = 0.0129). Other indexes like SD, CV, MAG, MAGE, and MODD did not show significant differences among groups in any of the three analyzed days (Table 4).

### 3.5. Effects of Treatments on Fasting Biochemical Parameters

NBS supplementation for four days induced a significant reduction in fasting glucose levels with respect to DMS (*p* < 0.05). However, no differences were found in other parameters such as insulin, triglycerides, cholesterol, GLP-1, and HOMA-IR (Table 5).

## 4. Discussion

In this study, we examined the effect of resistant starch from two sources administered over a period of four days on GC and GV in patients with T2D when all treatments were matched by rapid digestible starch content. We found that treatments containing 40 g of RS did not improve GC or GV.

Our original hypothesis was that RS treatments would induce beneficial effects on glycemic metabolism independently of the digestible starch content. We assumed that other possible mechanisms such as its property to modulate intestinal microbiota, appetite hormones, viscosity, or gastric emptying could play a role [21,22]. Contrary to our hypothesis, RS did not induce a reduction in the 24 h MBG levels, but it actually enhanced them in comparison with the DMS in the period from days 2 to 4. These findings disagree with a previous study from our team, where 38.3 g of NBS administered during four days reduced the 48 h glycemia AUC in comparison with the same doses of digestible corn starch in lean persons and patients with obesity [13]. The remarkable difference between these studies is that, in the one performed earlier, the two administered treatments were unmatched for digestible starch content and, therefore, the beneficial effect of NBS on MBG was expected owing to the difference in digestion rates between starches. Here, all treatments were matched by digestible starch content to receive 26.6 g of DS/day. It is well known that the digestion rate is the key mechanism for regulating postprandial blood glucose response and depends on the amylolysis caused by the gastrointestinal enzymes. It is worth mentioning that most studies encountering improved glycemic outcomes after RS supplementation have been conducted in experiments where RS replaces DS content [13,23,24] and, in contrast, when RS is added as an extra portion to DS content the results have been mixed [7,25].

Taking the effects on GV into consideration, no statistical differences were observed between groups during days 2 and 3. Nevertheless, on day 4, CONGA, GRADE, and J-index values were higher in HMS than in DMS, while other indexes such as SD, CV, MAG, MAGE, and MODD did not reach statistical significance. These results could be associated with the cumulative effects of the previous three days of supplementation and the poor GC of the patients. To the best of our knowledge, there is not another short-term study analyzing the effects of RS on GV that also matched the digestible starch content. However, in a recent study, a high-SDS diet induced a reduction on GV indexes in participants with well-controlled T2D. In that study, the SDS food content was evaluated using the software Nutrilog^®^ and the comparator was a group of participants consuming a diet with a low SDS content [26]. Thus, similar to studies where RS or SDS replaces digestible starch content, the beneficial effects of RS can be expected owing to the reduction in postprandial glycemia, which in turn is associated with reduced GV parameters [27,28].

Regarding fasting parameters, no modifications were observed after four days of RS treatments; the sole exception was that NBS treatment induced a reduction in fasting glycemia on day 5. This finding is in accordance with a longer study where 30 g/day of NBS was administered to women with obesity during 8 weeks [11]; however, it is in contrast with a different one where 24 g/day of the same product was given to women with diabetes over 4 weeks [10]. In addition, in other study providing 40 g/day of high-amylose corn starch for 12 weeks to patients with T2D, no changes in fasting glycemia or insulin were observed [29]. Comparisons with the present study, however, are difficult owing to the different intervention periods and doses.

Even though there is not an exact explanation for the unexpected effects of RS on GC and GV, it is important to consider that one of the most important mechanisms explaining the beneficial effect of RS is based on its property to be fermented by microbiota in the colon and subsequently generate short-chain fatty acids (SCFAs). These substances stimulate intestinal L-cells to produce hormones such as GLP-1 and PYY, which in turn modulate glucose uptake and appetite. In the present study, however, no effects of NBS supplementation were observed in fasting GLP-1 changes in comparison with DMS after four days of treatment. According to previous studies, only 1 to 3 days of supplementation are sufficient to induce a fermentation process in the colon [30,31,32].

Interestingly, other authors have suggested no improvements in GC after 12 weeks of prebiotic supplementation in patients with T2D, even though the bacterial diversity was increased [33]. This negative effect was also reported after 12 weeks of 40 g RS administered to patients with T2D [29]. Another group suggested that 45 g of high-amylose maize provided to adults with prediabetes for 12 weeks did not improve GC or other cardiovascular disease risk factors [34]. In addition, some studies have informed deleterious effects of fermentable carbohydrates on glucose homeostasis. RS intake has been reported to increase trimethylamine N-oxide, which is associated with increased risks of cancer, heart disease, and insulin resistance [35]. A different study informed that high doses of gluco- and galactooligosaccharides induced adverse effects on glucose response and fasting glycemia even when Bifidobacterium was increased [36]. Therefore, these findings along with ours highlight the importance of analyzing other factors affecting the effects of these substances on glycemic metabolism such as the baseline gut microbiota and drugs’ consumption.

The metabolic alterations in patients with T2D are, on the other hand, complex. It is known that these patients, in addition to a compromised β-cell function, also show a disruptive incretin system and an altered gut environment. Several studies have shown that these patients exhibit an increased permeability of butyrate secreted by intestinal epithelial cells conditioning the loss of the tight barrier function of intestinal cells, which induce endotoxemia and a low-grade inflammation state [37]. Additionally, it is known that metformin has a significant effect on microbiota composition, and sulfonylureas have been associated with an increased GV [37,38]. In the present study, the participants were receiving these drugs for GC; nonetheless, we consider that the influence of this potential confounding variable was reduced by the chosen crossover design, as all patients were maintained with the same medication along with the different treatment phases.

Another way to explain our results may be the underlying dietary variability due to the lack of adherence to the diet recommendations during the treatment period. In other words, even when similar reports of energy and macronutrients were informed by the participants, unreported alterations in their dietary pattern cannot be discarded. Another factor that could have influenced our results is the different composition of the beverages used to dilute the treatments, as the participants of this study were free to dilute supplements in any liquid of their choice. Thus, some of them reported having used pure water, cow’s milk, fruit juices, or regional beverages. In this way, the interaction with other macronutrients such as protein and fat could have affected the response of NBS, as it is known that these substances can affect the rate of amylolisis and glucose absortion [39]. In this sense, an appropriate regulation of the type of beverage as well as meal times is a very important aspect to take into consideration in future studies. Another possible reason behind our findings may be the dose calculation method used. Here, calculations to determine the digestible starch content in HMS and DMS were based in the specified quantities of fractions of starch (RDS, SDS, and RS) informed by the manufacturers. Even when this procedure has been widely used by different research groups [25,29,40,41,42], our best recommendation is to confirm the starch fractions of the treatments by measuring the in vitro digestibility at the laboratory.

The findings from this study should be interpreted with caution owing to the metabolic characteristics of the participants, the short-term supplementation, the lack of a direct supervision of RS consumption, and the absence of measurements of the in vitro digestibility rate of the commercial products. However, some advantages of the present work included the use of a crossover design in which participants constituted their own control, thereby decreasing within-participant variation and enabling a smaller sample size. The use of the CGM system has long been considered the best tool to estimate glucose excursions in a real-world setting. In addition, the daily caloric intake and exercise pattern of participants were controlled during the experiment.

## 5. Conclusions

In conclusion, in patients with T2D and uncontrolled glycemia, 4 days of RS supplementation did not show beneficial effects on GC or GV when all treatments were matched for digestible starch content. Our results do not support the hypothesis that the beneficial effects of RS are independent of its ability to lower blood glucose levels through a reduction in the amount of available carbohydrates. Future longer studies are needed to determine whether these findings were affected by a different baseline microbiota, underlying dietary variability, or other environmental factors.

## Figures and Tables

**Table 1:** Baseline characteristics of the study participants.

Characteristic	Total
Subjects (*n*)	10
Sex, M/F [*n* (%)]	5/5 (50.0/50.0)
Age (years)	48.5 ± 9.12
Weight (kg)	77.32 ± 11.58
BMI (kg/m^2^)	29.05 ± 3.84
HbA1c (%)	9.58 ± 2.13
Fasting glycemia (mg/dL)	203 ± 77.22
Insulin (μUI/mL)	7.4 ± 4.74
Triglycerides (mg/dL)	148 (103.3, 266.8)
Cholesterol (mg/dL)	189 ± 27.38
Medication	
Only metformin (*n*)	4
Metformin + Glibenclamide (*n*)	6

Data are expressed as mean ± SD or median (25th, 75th percentiles). BMI, body mass index.

**Table 2:** Daily dietary intake during each supplementation period.

Ingredients	Treatments			*p*
	DMS	HMS	NBS	
Energy (kcal)	1711 ± 77.87	1558 ± 57.19	1576 ± 106	0.1135
Carbohydrates (g)	198.9 ± 9.15	215.7 ± 14.34	194.7 ± 13.89	0.2691
Proteins (g)	63.28 ± 2.65	69.53 ± 6.23	64.18 ± 3.24	0.2030
Fat (g)	54.35 ± 1.96	64.65 ± 11.17	59.58 ± 7.38	0.4405
Fiber (g)	13.8 ± 0.98	14.58 ± 1.41	12.65 ± 1.78	0.1358

Data are expressed as mean ± SD of daily dietary intake during the four days of supplementation without considering treatment content. The *p*-values were obtained from comparisons based on two-way repeated measures ANOVA and Tukey’s post hoc test (*n* = 10).

**Table 3:** Effects of treatments on glycemic control during days 2 to 4.

GC Indexes	Period (Days)	Treatments		
		DMS	HMS	NBS
24 h MBG	2	164.2 (151.3, 177.9)	180.2 (166.7, 193.8) ^a^	178.7 (166.4, 187.6) ^b^
	3	163.9 (154.7, 167.2)	183.6 (175.8, 190.1) ^ac^	173.3 (170.6, 177.1) ^bc^
	4	151.3 (144.5, 162.1)	189.2 (173.5, 200.8) ^ac^	172.8 (164.1, 179.2) ^bc^
Maximum glucose	2	241.4 ± 90.08	249.8 ± 85.25	243.7 ± 68.60
	3	230.8 ± 83.48	244.3 ± 86.98	228 ± 71.13
	4	213.2 ± 83.81	260.4 ± 80.31	233 ± 82.19
Minimum glucose	2	93.50 (84.50, 119.3)	108 (88.25, 211)	110 (94.75, 181.3)
	3	105.5 (85.76, 147.5)	106.5 (85.50, 218.5)	96 (89.75, 143.8)
	4	91 (73.75, 129)	106 (81, 217)	93.50 (83, 199.8)
HBGI	2	3.55 (0.97, 15.61)	3.62 (1.20, 29.35)	3.71 (2.25, 18.81)
	3	3.03 (1.51, 14.07)	4.82 (1.09, 27.99)	3.19 (1.54, 16.28)
	4	3.17 (0.49, 9.79)	5.37 (2.20, 24.16) ^a^	2.51 (1.25, 18.69)
LBGI	2	0.404 (0.002, 0.818)	0.028 (0.0, 0.606)	0.005 (0.0, 0.465)
	3	0.061 (0.0, 0.687)	0.061 (0.0, 0.915)	0.392 (0.0, 0.641)
	4	0.352 (0.0, 1.20)	0.059 (0.0, 0.836)	0.453 (0.0, 0.775)
LAGE	2	128.1 ± 61.42	115.5 ± 44.94	115 ± 45.22
	3	114.2 ± 53.42	111.7 ± 50.66	109.6 ± 32.20
	4	109.1 ± 56.11	129.5 ± 52.56	99.80 ± 31.45
TAR (%)	2	14.24 (0.0, 59.20)	12.50 (0.00, 100.4)	11.63 (4.42, 99.57)
	3	6.94 (0.00, 62.07)	17.71 (0.00, 100.4)	9.37 (2.60, 94.10)
	4	7.98 (0.00, 61.02)	25.52 (1.82, 100.4)	48.09 (3.12, 100.4)
TBR (%)	2	0	0	0
	3	0	0	0
	4	0	0 (0.00, 0.35)	0
TIR (%)	2	81.95 (21.79, 100.4)	88.03 (0.00, 100.4)	88.72 (0.00, 96.96)
	3	84.03 (38.20, 100.4)	81.77 (0.00, 100.4)	90.97 (6.24, 97.75)
	4	92.36 (39.33, 100.4)	74.83 (0.00, 95.40)	0 (0.00, 100.3)

Data are expressed as mean ± SD or median (25th and 75th percentiles). Differences were based on one-way repeated measures ANOVA or Friedman test (*n* = 10). ^a^ *p* < 0.05 HMS vs. DMS; ^b^ *p* < 0.05 NBS vs. DMS; ^c^ *p* < 0.05 HMS vs. NBS. DMS, digestible maize starch; HMS, Hi-Maize starch; NBS, native banana starch; 24 h MBG, 24 h mean blood glucose; HBGI, high blood glucose index; LBGI, low blood glucose index; LAGE, large amplitude of glycemic excursions; TAR, time above range; TBR, time below range; TIR, time in range.

**Table 4:** Effects of treatments on glycemic variability during days 2 to 4.

GV Indexes	Period (Days)	Treatments		
		DMS	HMS	NBS
SD	2	1.80 ± 1.04	1.54 ± 0.75	1.57 ± 0.75
	3	1.57 ± 0.82	1.49 ± 0.69	1.35 ± 0.46
	4	1.32 (0.68, 1.91)	1.68 (1.23, 2.55)	1.18 (0.79, 1.41)
CV	2	18.75 ± 8.46	16.01 ± 6.99	16.27 ± 6.37
	3	17 ± 6.76	15.12 ± 6.44	14.23 ± 3.14
	4	15.88 ± 5.95	18.14 ± 7.74	13.30 ± 3.84
CONGA	2	8.56 ± 3.49	9.16 ± 3.97	9.13 ± 3.31
	3	8.30 ± 3.07	9.34 ± 3.90	8.88 ± 3.47
	4	6.65 (5.88, 9.98)	7.44 (6.47, 13.58) ^a^	6.84 (5.94, 12.37)
GRADE	2	7.29 ± 7.57	9.46 ± 9.41	9.67 ± 8.50
	3	7.34 ± 7.19	10.19 ± 9.27	9.03 ± 8.27
	4	6.65 ± 7.08	10.80 ± 8.85 ^a^	8.78 ± 8.55
MAG	2	1.18 ± 0.45	1.33 ± 0.48	1.27 ± 0.32
	3	1.16 ± 0.48	1.33 ± 0.41	1.22 ± 0.32
	4	1.11 ± 0.51	1.41 ± 0.49	1.19 ± 0.33
J-Index	2	44.61 ± 35.78	49.36 ± 39.65	46.73 ± 30.27
	3	40.45 ± 29.51	49.79 ± 37.72	43.42 ± 31.47
	4	27.90 (16.53, 45.60)	35.76 (24.36, 82.68) ^a^	25.32 (20.24, 66.64)
MAGE	2	4.84 ± 2.53	4.05 ± 1.54	4.23 ± 1.90
	3	4.04 (1.59, 5.57)	3.83 (1.99, 6.09)	3.36 (2.57, 4.27)
	4	3.62 ± 2.11	4.78 ± 1.81	3.41 ± 1.57
MODD	2–4	1.73 ± 0.80	1.75 ± 0.89	1.57 ± 0.58

Data are expressed as mean ± SD or median (25th and 75th percentiles). Differences were based on one-way repeated measures ANOVA or Friedman test (*n* = 10). ^a^ *p* < 0.05 HMS vs. DMS. DMS, digestible maize starch; HMS, Hi-Maize starch; NBS, native banana starch; CV, coefficient of variation; CONGA, continuous overall net glycemic action; GRADE, glycemic risk assessment in diabetes equation; MAG, mean absolute glucose; MAGE, mean amplitude of glycemic excursions; MODD, mean of daily differences.

**Table 5:** Effects of resistant starch on fasting biochemical parameter changes from baseline.

Parameter	Treatments			*p*
	DMS	HMS	NBS	
Glucose (mg/dL)	−14.41 ± 32.35	−1.00 ± 34.47	−36.47 ± 36.86	0.0074 ^b^
Insulin (µUI/mL)	−4.0 (−7, 0.5)	−1.0 (−6.0, 4.0)	0.0 (−3.5, 1.5)	0.3837
Triglycerides (mg/dL)	−11.00 (−490.0, 18.00)	−32.0 (−98.5, −5.5)	−24.00 (−389.0, 13.0)	0.9752
Cholesterol (mg/dL)	−4.94 ± 32.99	−0.94 ± 33.55	−10.76 ± 44.81	0.7125
GLP-1 (pM/L)	−4.496 ± 15.03	^£^	5.379 ± 22.09	0.2344
HOMA-IR	−1.91± 2.2	0.28 ± 5.77	−1.88 ± 3.03	0.1167

Data correspond to differences in fasting value changes from baseline between day 5 and day 1 in patients with T2D (*n* = 17). Data are expressed as mean ± SD or median (25th and 75th percentiles). Comparisons were based on one-way ANOVAs in combination with Tukey’s tests or Student’s *t*-tests. ^b^ *p* < 0.05 NBS vs. DMS. HOMA-IR, homeostatic model to evaluate insulin resistance; GLP-1, glucagon-like peptide-1. ^£^ Data not obtained.

## Data Availability

The dataset used in this publication is available from the corresponding author on reasonable request.

## Supplementary Materials

The following are available online at https://www.mdpi.com/article/10.3390/nu13114052/s1, Figure S1: Effects of treatments containing resistant starch on glycemic excursions determined by CGM system during day 2 (**A**), day 3 (**B**), and day 4 (**C**); Figure S2: Effects of treatments containing resistant starch on incremental glycemic excursions determined by CGM system during day 2 (**A**), day 3 (**B**), and day 4 (**C**); Figure S3: Effects of treatments containing resistant starch on glycemic excursions during day 4.
