# Supplementary material for: Resistant Starch Consumption Effects on Glycemic Control and Glycemic Variability in Patients with Type 2 Diabetes: A Randomized Crossover Study
Source: Nutrients. 2021 Nov 12;13(11):4052. doi: 10.3390/nu13114052 (PMC8621288; doi:10.3390/nu13114052)
Supplement: Supplementary file 1 [file nutrients-13-04052-s001.zip › nutrients-1418902-supplementary.pdf]

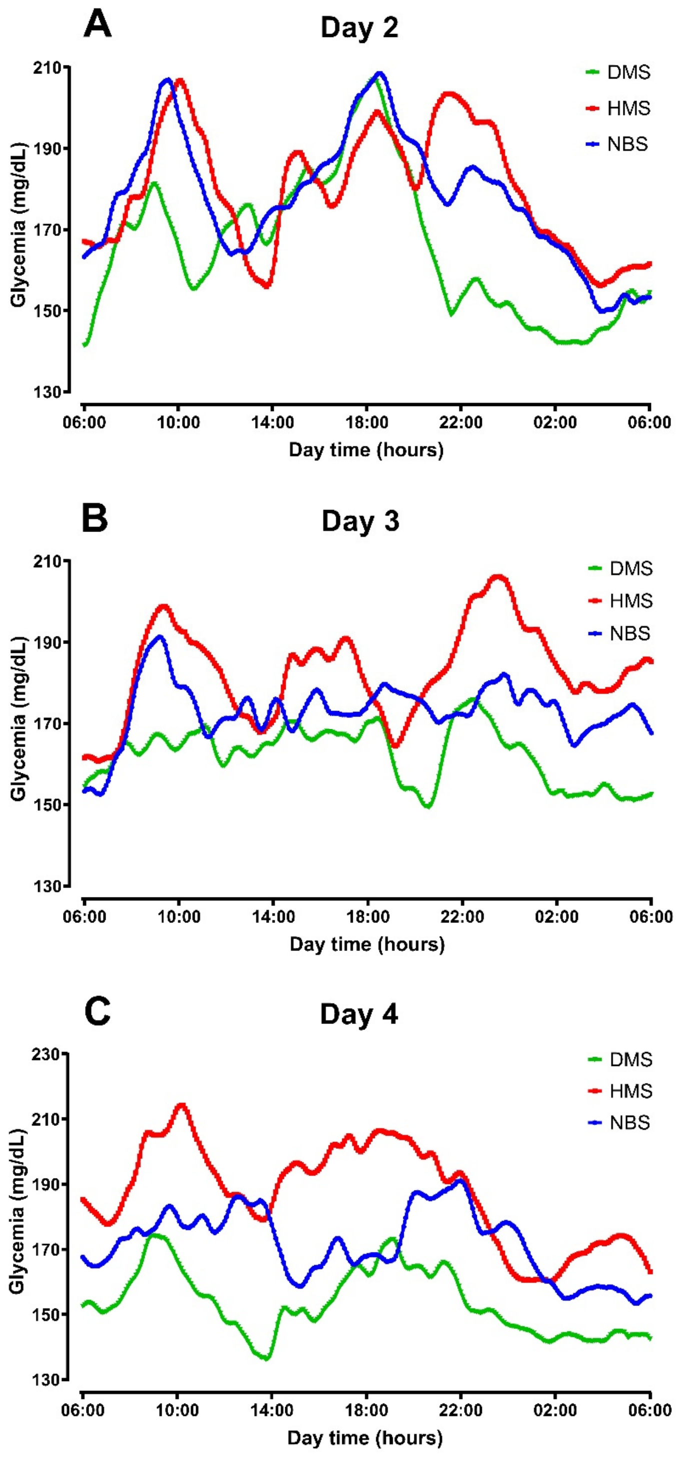

Figure S1. Effects of treatments containing resistant starch on glycemic excursions determined by CGM system during day 2 (**A**), day 3 (**B**), and day 4 (**C**). Data are expressed as only means of ten patients. Comparisons are based on Friedman test. DMS, Digestible Maize Starch; HMS, Hi Maize Starch; NBS, Native Banana Starch.

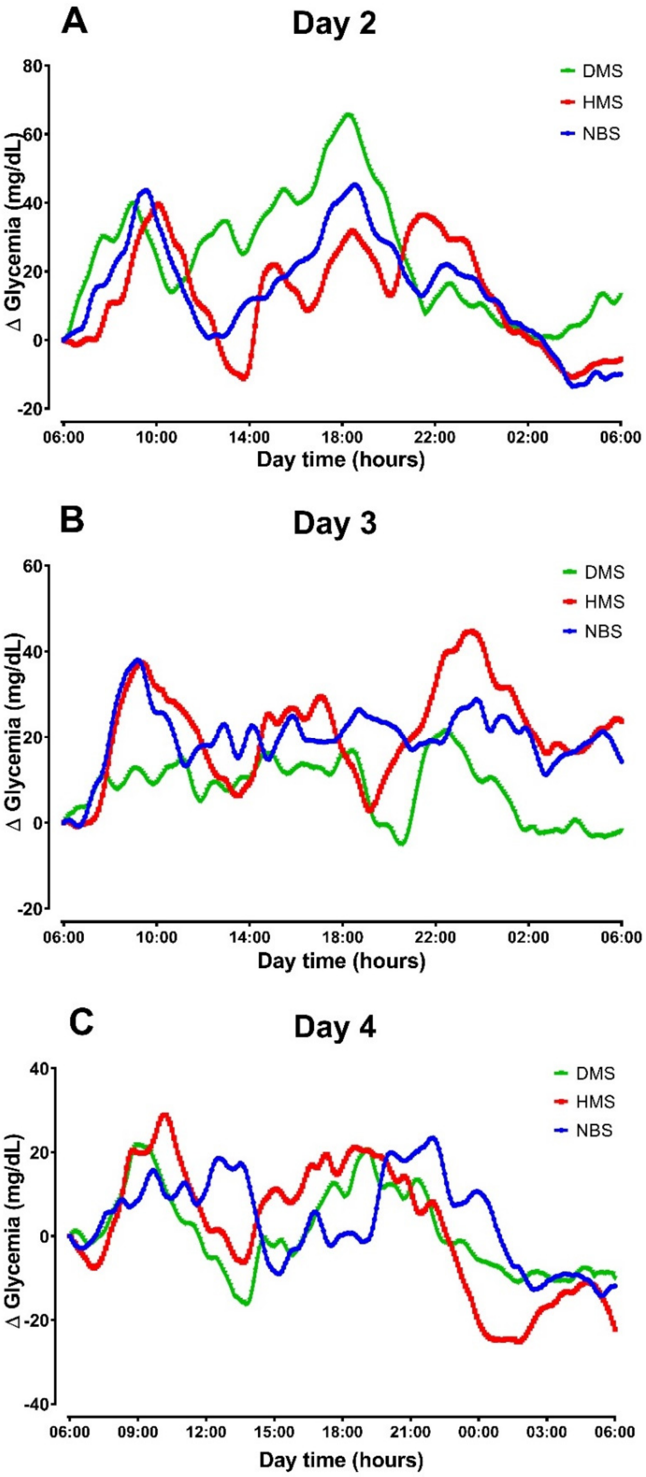

Figure S2. Effects of treatments containing resistant starch on incremental glycemic excursions determined by CGM system during day 2 (**A**), day 3 (**B**), and day 4 (**C**). Data are expressed as only means of ten patients. Comparisons are based on Friedman test. DMS, Digestible Maize Starch; HMS, Hi Maize Starch; NBS, Native Banana Starch.

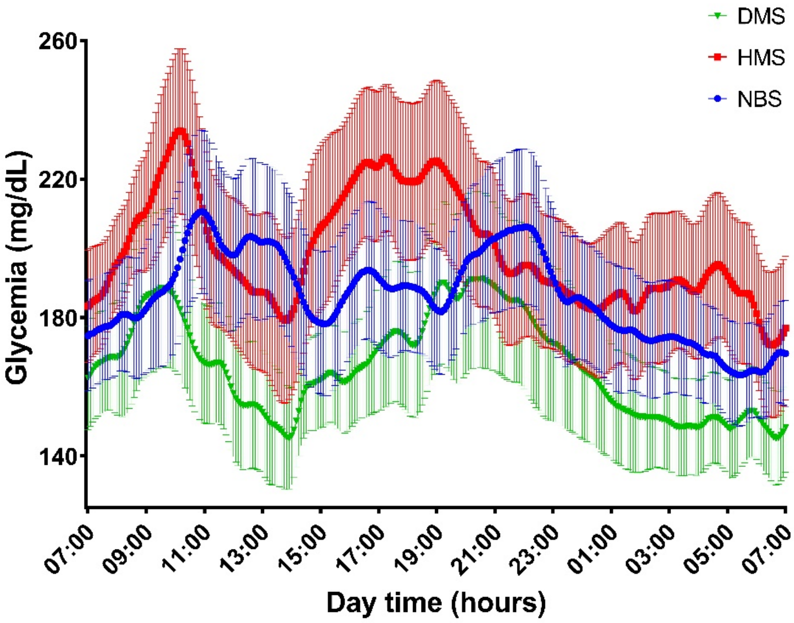

Figure S3. Effects of treatments containing resistant starch on glycemic excursions during day 4. Data are expressed as means and SD of ten patients. Comparisons are based on one-way ANOVA and the Tukey´s post-hoc test. DMS, Digestible Maize Starch; HMS, Hi Maize Starch; NBS, Native Banana Starch.
